# High‐Resolution Diffusion‐Weighted Imaging With Self‐Gated Self‐Supervised Unrolled Reconstruction

**DOI:** 10.1002/mrm.70250

**Published:** 2026-01-22

**Authors:** Zhengguo Tan, Patrick A. Liebig, Annika Hofmann, Frederik B. Laun, Florian Knoll

**Affiliations:** ^1^ Michigan Institute for Imaging Technology and Translation (MIITT), Department of Radiology University of Michigan Ann Arbor Michigan USA; ^2^ Ultra‐High Field Predevelopment Team Siemens Healthcare GmbH Erlangen Bavaria Germany; ^3^ Artificial Intelligence in Biomedical Engineering Friedrich‐Alexander‐University Erlangen‐Nuremberg Erlangen Bavaria Germany; ^4^ Institute of Radiology University Hospital Erlangen Erlangen Bavaria Germany

**Keywords:** algorithm unrolling, diffusion weighted imaging, image reconstruction, machine learning, self‐supervised learning, submillimeter resolution

## Abstract

**Purpose:**

High‐resolution diffusion‐weighted imaging (DWI) is clinically demanding. The purpose of this work is to develop an efficient self‐supervised algorithm unrolling technique for submillimeter‐resolution DWI.

**Methods:**

We developed submillimeter DWI acquisition utilizing multi‐band multi‐shot EPI with diffusion shift encoding. We unrolled the alternating direction method of multipliers (ADMM) to perform scan‐specific self‐gated self‐supervised DeepDWI learning for multi‐shot echo planar imaging with diffusion shift encoding on a clinical 7 T scanner.

**Results:**

We demonstrate that (1) ADMM unrolling is generalizable across slices, (2) ADMM unrolling outperforms multiplexed sensitivity‐encoding (MUSE) and compressed sensing with locally‐low rank (LLR) regularization in terms of image sharpness, tissue continuity, and motion robustness, and (3) ADMM unrolling enables clinically feasible inference time.

**Conclusion:**

Our proposed ADMM unrolling enables whole brain DWI of 21 diffusion volumes at 0.7 mm isotropic resolution and 10 min scan, and shows higher signal‐to‐noise ratio (SNR), clearer tissue delineation, and improved motion robustness, which makes it plausible for clinical translation.

## 1 Introduction

Diffusion‐weighted imaging (DWI) [1] has been an important imaging modality in neuro‐scientific research and clinical diagnosis and staging of tumors. However, clinical DWI, based on single‐shot echo planar imaging (EPI) [2], poses challenges in the pursuit of high spatial, temporal, and angular resolution. Until now, the search for precise neuro imaging has fostered significant advances in DWI, including multi‐shot EPI (interleaved [3, 4, 5] and readout‐segmented [6, 7]), field inhomogeneity and eddy current correction [8], simultaneous multi‐slice [9], reconstruction techniques such as parallel imaging [10, 11, 12] and compressed sensing [13, 14, 15], as well as diffusion‐weighted image denoising [16, 17].

In the area of parallel imaging and compressed sensing, low rankness has been powerful prior knowledge for regularized iterative reconstruction. Mani et al. [14] developed MUlti‐Shot Sensitivity Encoded Diffusion Data Recovery using Structured Low Rank Matrix Completion (MUSSELS), in which structural low rankness is enforced in multi‐shot k‐space. After reconstruction, one diffusion‐weighted image is calculated via root sum of square of all shot images. MUSSELS bypasses the shot‐to‐shot phase variation correction. Hu et al. [15] developed magnitude‐based spatial‐angular locally low‐rank regularization (SPA‐LLR), which employs locally‐low rank (LLR) regularization [18] in joint k‐q‐space reconstruction. Tan et al. [19] extended LLR to accelerated multi‐band multi‐shot multi‐shell reconstruciton. Dong et al. [20] proposed to better enforce the spatial‐diffusion matrix low rankness by stacking shuffled patches together. However, low rank type iterative methods require long reconstruction time, due to the computation of singular value decomposition on many local patches.

Recently, algorithm unrolling emerges as an interpretable and efficient deep learning technique for signal and image processing [21]. In the context of iterative image reconstruction, algorithm unrolling naturally inherits domain knowledge, that is, the physics‐based forward modeling and the data consistency term. Therefore, algorithm unrolling does not need to learn that domain knowledge from intensive training data. Furthermore, algorithm unrolling can replace the regularization term in compressed sensing with deep neural networks, acting as an implicit regularizer in inverse problems. Traditional iterative image reconstruction represents the regularization term as one linear transformation followed by one nonlinear thresholding function. For instance, the low rankness regularization is done by singular value soft thresholding (nonlinear) on Casorati matrices. In contrast, deep neural networks are constructed by multiple layers comprising distinct linear transformations and nonlinear activation functions. Therefore, algorithm unrolling offers high representation power. There exist pioneering works that leverage algorithm unrolling for accelerated parallel imaging reconstruction [22, 23, 24, 25].

Algorithm unrolling has been introduced to DWI image reconstruction. Mani et al. [26] proposed to learn a denoising autoencoder (DAE) model [27] from a dictionary simulated from the ball‐and‐stick model. This learned DAE model is subsequently utilized as a q‐space regularizer, in combination with a total‐variation spatial regularizer, in the joint k‐q‐space reconstrution. Although promising, this method is specific to diffusion tensor models and may not generalize well to DWI image reconstruction. Alternatively, self‐supervised learning that requires neither large‐scale dictionaries nor high‐quality fully‐sampled reference data has been explored. Based on the zero‐shot learning concept [28], Cho et al. [29] proposed to learn a k‐space regularization function for multi‐shot DWI reconstruction. This approach learns an unrolled algorithm utilizing only the acquired data itself, and thus requires no extra training data and is scan specific. Similar to MUSSELS, this approach addresses the multi‐shot EPI reconstruction problem for a single diffusion encoding, and does not perform joint reconstruction that explores q‐space redundancy. Further, its effectiveness for high‐resolution DWI is yet to be explored.

To achieve submillimeter isotropic DWI at clinically feasible scan and inference times, we propose a novel solution that leverages an ADMM unrolling method with self‐supervised learning for multi‐band, multi‐shot DWI with diffusion shift encoding. Our approach incorporates complementary *k*‐*q*‐space sampling and jointly reconstructs multiple slices and all diffusion‐weighted images. Moreover, building upon the foundation of noise2noise [28, 30, 31], we train the ADMM unrolling model using a single multi‐band slice, thereby enabling self‐gated joint reconstruction with significantly reduced training time and without the need for large‐scale dictionaries or extra training data. Our method not only achieves high‐resolution DWI at 0.7 mm isotropic resolution with 21 diffusion‐encoding directions but also does so in under 10 min of scan time and approximately 1 min of reconstruction time per slice. This provides a clinically viable, efficient solution to the submillimeter resolution DWI challenge.

## 2 Methods

### 2.1 In Vivo Acquisition and Reconstruction

Table 1 lists two acquisition protocols implemented on a clinical 7 T MR system (MAGNETOM Terra, Siemens Healthineers, Erlangen, Germany) equipped with a 32‐channel head coil (Nova Medical, Wilmington, MA, USA) and the XR‐gradient system (maximum gradient strength 80 mT/m and a peak slew rate 200 T/m/s). Protocol #1 with 1 mm isotropic resolution serves as the reference with in‐plane fully sampling and the multi‐band factor 3. This reference 4‐shot data is retrospectively undersampled to only 2 shots (i.e., 2‐fold in‐plane undersampling) and then trained and tested with the proposed self‐supervised learning. Protocols #2 and #3 realize high resolution mesoscale DWI with isotropic resolution 0.7 mm. Two‐fold acceleration is used in both the in‐plane and in‐slice directions. Every diffusion encoding is acquired by three shots in an interleaved manner and is shifted with respect to its former, resulting in a 6×2‐fold acceleration per shot. It is noteworthy that the total scan time can be reduced to about 10 min (Protocol #3) when switching off navigator acquisition.

**TABLE 1:** Acquisition protocols.

Protocol^a^	#1 (1.0 mm)	#2 (0.7 mm NAV)	#3 (0.7 mm)
FOV (mm^2^)	200		
Matrix size	200×200×114	286×286×176	
Voxel (mm^3^)	1.0×1.0×1.0	0.7×0.7×0.7	
Shots	4	3	
Acceleration	1×3	2×2	
Partial Fourier	5/8	5/8	
Bandwidth (Hz/Pixel)	1086	972	
ESP (ms)	1.04	1.17	
Navigator	No	Yes	No
TE (ms)	66	58/98.3	58
TR (ms)	5400	15 000	8900
Acquisition (min)	7:52	16:27	9:57

^a^ All protocols employed the MDDW diffusion acquisition mode with monopolar diffusion encoding gradients, 1 $b_{0}$ volume and 20 diffusion‐weighted volumes with the b‐value of 1000s/mm^2^.

Three young healthy volunteers with written informed consent approved by the local ethics committee participated in this study. All reconstructions in this work were done on a single A100 SXM4/NVLink GPU with 80 GB memory (NVIDIA, Santa Clara, CA, USA).

### 2.2 Multi‐Band Multi‐Shot DWI With Diffusion‐Shift Encoding

**FIGURE 1:**
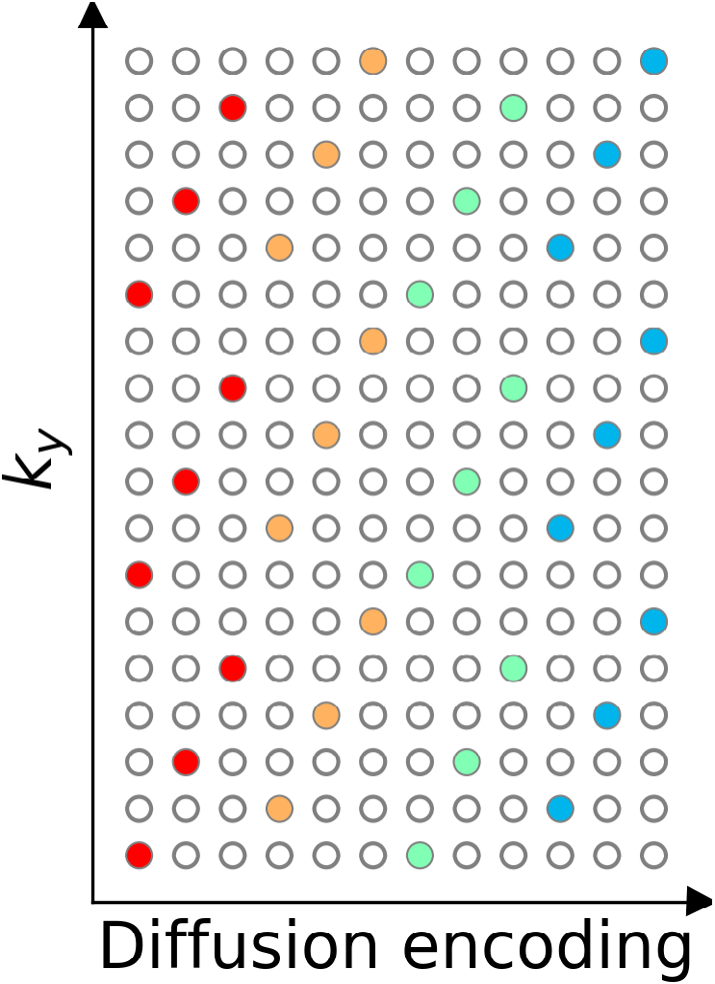
Three‐shot DWI with diffusion shift encoding. This work employs three‐shot per diffusion encoding and each shot has an in‐plane undersampling factor of 6. Every three columns assemble one diffusion encoding and thus are colored the same. The starting $k_{y}$ line is shifted between adjacent diffusion encoding to create complementary k‐q‐space sampling.

Our previous work [19] demonstrated the joint k‐q‐slice reconstruction for multi‐band multi‐shot navigator‐based interleaved EPI (NAViEPI) DWI acquisition with diffusion shift encoding. As shown in Figure 1, the starting line $k_{y}$ for a diffusion encoding is shifted with respect to its adjacent line to create a complementary k‐q‐slice sampling pattern. In the joint reconstruction, the forward model maps the multi‐slice multi‐diffusion‐weighted images (x) to their corresponding k‐space,

(1) 𝒜(x)=P∑ΘFSΦx

Here, the images x are point‐wise multiplied with the precomputed shot‐to‐shot phase variation maps (Φ) and coil sensitivity maps (S). The output images are then converted to k‐space via the two‐dimensional fast Fourier transform (F), multiplied point‐wise with the multi‐band phases (Θ), summed along the slice dimension (∑), and then multiplied by the k‐space undersampling mask (P).

In Equation (1), one challenge is to accurately estimate the shot‐to‐shot phase variation. Multiplexed sensitivity‐encoding (MUSE)‐type reconstruction techniques [4, 5, 32, 33] achieved the self‐gating strategy, where the *k*‐space data of each shot were used to reconstruct its corresponding shot image followed by a phase smoothing approach (i.e., the phase variation operator Φ). Self‐gated shot phase estimation does not require the acquisition of phase navigator data, thereby rendering short scan time. In the imaging scenario of submillimeter resolution, usually many shots are needed. As a result, this increases the acceleration factor per shot and thus necessitates the use of navigators. The drawback of adding navigators is the increase of scan time. Therefore, this work aims to develop an efficient DWI protocol that can achieve submillimeter resolution while retaining short scan time.

With the operator 𝒜, the joint reconstruction reads,

(2) $$\underset{x}{\text{argmin}}{\left\|y-𝒜\left(x\right)\right\|}_{2}^{2}+\lambda ℛ\left(x\right)$$

where y is the measured k‐space data. The first term in Equation (2) presents the data‐consistency term, and the second term presents the regularization function ℛ(x) with the regularization strength λ. When using the Tikhonov regularization, that is, $ℛ\left(x\right)={\left\|x\right\|}_{2}^{2}$, Equation (2) can be solved via the conjugate gradient (CG) method. For nonlinear regularization functions, such as the locally‐low rank (LLR) regularization [19] or neural networks with nonlinear activation functions. ADMM was implemented in PyTorch to solve for Equation (2).

### 2.3 Image Reconstruction via Self‐Supervised ADMM Unrolling

Instead of the two‐step alternating minimization unrolling scheme as used in MoDL [25], we employed the ADMM unrolling to solve the self‐supervised learning reconstruction in Equation (2). The update rule of ADMM unrolling reads:

(3) $$\left\{\begin{array}{l} x^{\left(k+1\right)}=\underset{x^{\left(k\right)}}{\text{argmin}}{\left\|y-𝒜\left(x^{\left(k\right)}\right)\right\|}_{2}^{2}\\ \;+\;\frac{\rho }{2}{\left\|x^{\left(k\right)}-v^{\left(k\right)}+u^{\left(k\right)}\right\|}_{2}^{2}\\ v^{\left(k+1\right)}=\left(\lambda /\rho \right)\cdot {𝒟}_{\omega }\left(x^{\left(k+1\right)}+u^{\left(k\right)}\right)\\ u^{\left(k+1\right)}=u^{\left(k\right)}+x^{\left(k+1\right)}-v^{\left(k+1\right)} \end{array}\right.$$

ADMM updates the variables x, v, and u in an alternating scheme. It splits the unrolled reconstruction into three steps, as shown in Equation (3) and in the pseudo code of Algorithm 1. First, the updating step for x is solved by conjugate gradient. Second, the variable v is then updated via the forward pass of the neural network ${𝒟}_{\omega }$ with the input as the sum of current estimates of x and u. Third, the variable u is updated by adding its current estimate to the difference between x and v.

ALGORITHM 1 **Self‐Supervised ADMM Unrolling.**

1: **Initialization**:

2: split sampling mask P into 12 repetitions, each of which consists of three disjoint sets T, L, and V

3: p←0 and $N_{\text{epoch}}\leftarrow 100$

4: ${𝒟}_{\omega }$ set as ResNet

5: ρ←0.05 and λ←0.05

6: ${\text{Loss}}_{\text{valid}}\leftarrow \inf$ and trace←0

7: **function** ADMM(mask)

8: ${𝒜}_{\text{mask}}\leftarrow$ set the mask in the forward operator 𝒜

9: $x^{\left(0\right)}\leftarrow {𝒜}_{\text{mask}}^{H}\left(y\right)$

10: $v^{\left(0\right)}\leftarrow x^{\left(0\right)}$ and $u^{\left(0\right)}\leftarrow 0$

11: k←0 and $N_{\text{unroll}}\leftarrow 8$

12: **while** $k<N_{\text{unroll}}$ **do**

13:  $x^{\left(k+1\right)}\leftarrow$ conjugate gradient with 6 iterations

14:  $v^{\left(k+1\right)}\leftarrow \left(\lambda /\rho \right)\cdot {𝒟}_{\omega }\left(x^{\left(k+1\right)}+u^{\left(k\right)}\right)$

15:  $u^{\left(k+1\right)}\leftarrow u^{\left(k\right)}+x^{\left(k+1\right)}-v^{\left(k+1\right)}$

16:  k←k+1

17: **end while**

18: **return** $x^{\left(k+1\right)}$

19: **end function**

20: **Training:**

21: **while** $p<N_{\text{epoch}}$ or trace≤12 **do**

22: $x_{t}\leftarrow \text{ADMM}\left(T\right)$

23: ${\text{Loss}}_{\text{train}}\leftarrow ℒ\left(\mathrm{Ly},{𝒜}_{L}\left(x_{t}\right)\right)$

24: update ω via ADAM

25: **Validation**:

26: $x_{t}\leftarrow \text{ADMM}\left(T\cup L\right)$

27: ${\text{Loss}}_{\text{temp}}\leftarrow ℒ\left(\mathrm{Vy},{𝒜}_{V}\left(x_{t}\right)\right)$

28: **if** ${\text{Loss}}_{\text{temp}}\leq {\text{Loss}}_{\text{valid}}$ **then**

29:  ${\text{Loss}}_{\text{valid}}\leftarrow {\text{Loss}}_{\text{temp}}$

30:  trace←0

31: **else**

32:  trace←trace+1

33: **end if**

34: **end while**

**FIGURE 2:**
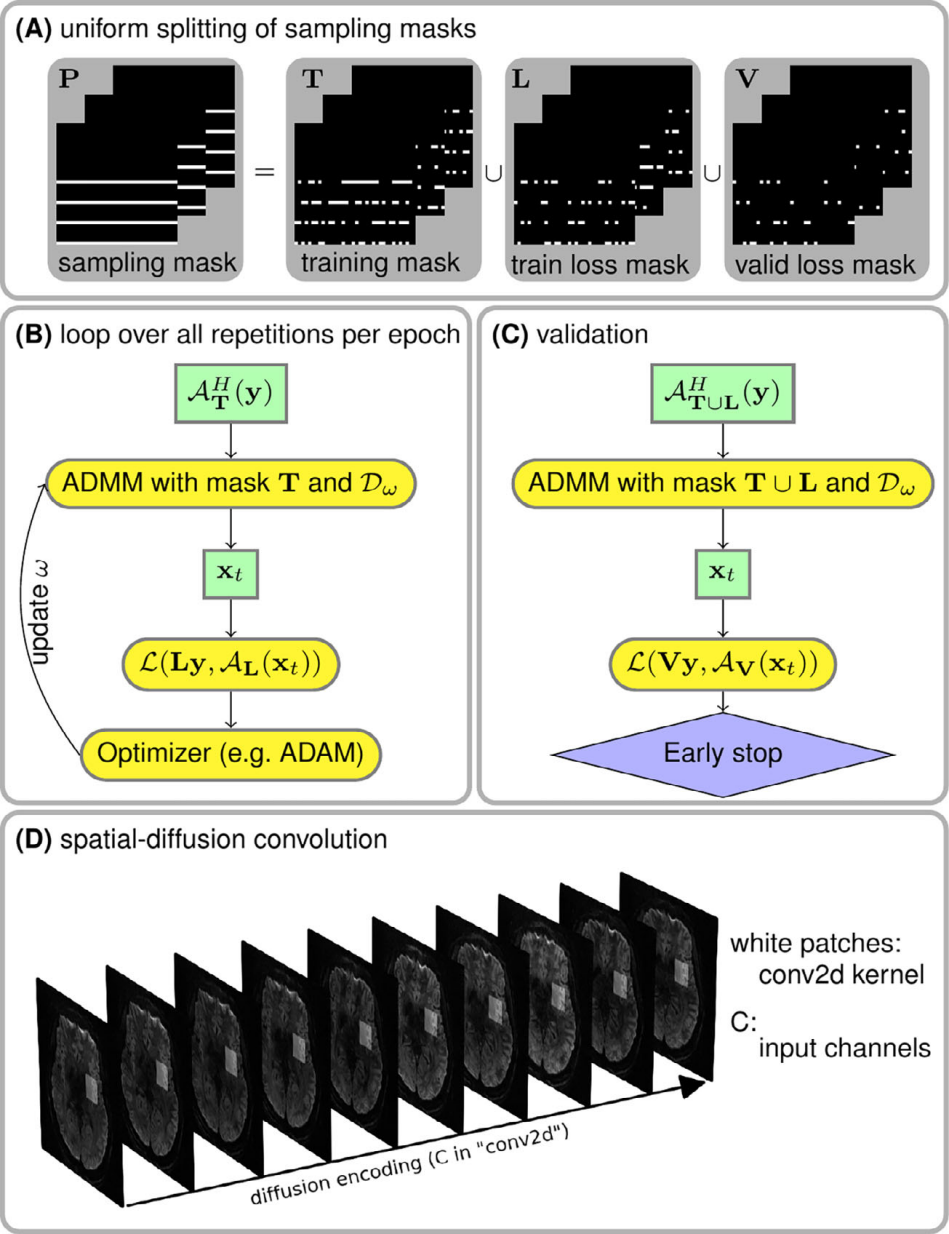
Illustration of the key components in ADMM unrolling. (A) The sampling mask P in Equation (1) was uniformly split into three disjoint sets: The training mask T used for the data consistency term during training, the train loss mask L used for the loss function calculation during training, and the validation loss mask V used for the loss function calculation during validation. (B) and (C) show the flowchart for the training and the validation of an unrolled ADMM model, respectively. Note that the ResNet parameters ω are updated via ADAM [34] during training, but remain fixed during the validation step. (D) A stack of diffusion‐weighted images is input into ResNet during ADMM unrolling.

Every training epoch consists of 12 looping repetitions. In each repetion, the data sampling mask P is split into three disjoint sets: the training mask T for the data consistency term, the training loss mask L for the loss function calculation, and the validation loss mask V, as shown in Figure 2. Each repetition has different masks. In each training epoch, the corresponding masks of the given repetition is used in order to update the ResNet parameters ω (Figure 2B). Plus, the validation step is performed after every training epoch to update the minimal validation loss. If the validation loss does not reduce for 12 consecutive epochs or if 100 epochs are reached, the training is terminated. The use of three disjoint masks is inline with the zero‐shot self‐supervised learning approach [28, 31] for scan‐specific parallel imaging reconstruction. In contrast, Self‐supervised learning via data undersampling (SSDU) [35] splits the sampling mask into only two sub‐masks, but requires multiple datasets for training.

The index k in Equation (3) denotes the unrolling iteration, and ${𝒟}_{\omega }$ denotes the ResNet [36] parameterized by ω (refer to Figure S1). In this work, 2D convolution was employed to construct the ResNet layers. In PyTorch, 2D convolution requires four‐dimensional tensors as input and output. For instance, a matrix with the size (N,C,H,W) is acceptable for the “conv2d” function in PyTorch. Here, W and H denote the width and height of the convolution kernel, C denotes the number of channels, and N denotes the batch size. However, the diffusion‐weighted images (x) to be reconstructed have the size $\left(N_{\text{diff}},N_{Z},N_{Y},N_{X},2\right)$, where 2 stands for the real and imaginary part of the complex‐valued diffusion‐weighted images, $N_{X}$ and $N_{Y}$ are the width and the height of diffusion‐weighted images, $N_{Z}$ is the number of slices (identical to the multi‐band factor), and $N_{\text{diff}}$ is the number of diffusion encoding. To train a ResNet based on 2D convolution, the diffusion‐weighted images were reshaped and permuted as $\left(N_{Z},2\cdot N_{\text{diff}},N_{Y},N_{X}\right)$, as illustrated in Figure 2D. In this manner, 2D convolution kernels in combination with ReLU activation functions loop through the varying diffusion‐weighted contrast to learn the key features of the high‐dimensional data and to reduce noisy and aliasing artifacts in unrolled reconstruction.

### 2.4 Model Generalizability

Volumetric whole brain DWI acquisition consists of many multi‐band slices, and the training of algorithm unrolling models on all slices requires hundreds of GPU computing hours. To investigate the model generalizability and to accelerate reconstruction, we performed two training and inference strategies. First, we trained the ADMM unrolling model with only one multi‐band slice data, and subsequently tested the model on all remaining multi‐band slices. We called this approach “single‐slice training”. Second, we trained and tested every multiband slice individually, which was referred to as “slice‐by‐slice training”. The single‐slice training strategy saves tremendous training time, as its model is learned from one single slice and the inference time per slice is only about 1 min. By comparing these two training strategies, we aim at demonstrating the model generalizability and its applicability to other slices which are “unseen” in single‐slice training.

### 2.5 Comparison of Regularization Techniques

This work compared the reconstruction performance of three different regularization techniques, Tikhonov $\ell^{2}$ regularization (as used in MUSE), LLR regularization, and ADMM unrolling with a learned regularization. Note that MUSE is a simultaneous multi‐slice (SMS) parallel imaging method and poses no regularization along the diffusion dimension, effectively solving each DWI reconstruction independently. In contrast, the other two regularized reconstructions fall into the joint reconstruction regime. They jointly reconstruct all diffusion‐weighted images and impose regularization terms exploring spatial‐diffusion redundancies. For example, LLR enforces the low rankness of local spatial‐diffusion matrices from diffusion‐weighted images, whereas ADMM unrolling learns a regularization function composed by neural networks based on spatial‐diffusion convolution kernels while enforcing data consistency during the unrolled training process.

## 3 Results

### 3.1 Retrospective Study

**FIGURE 3:**
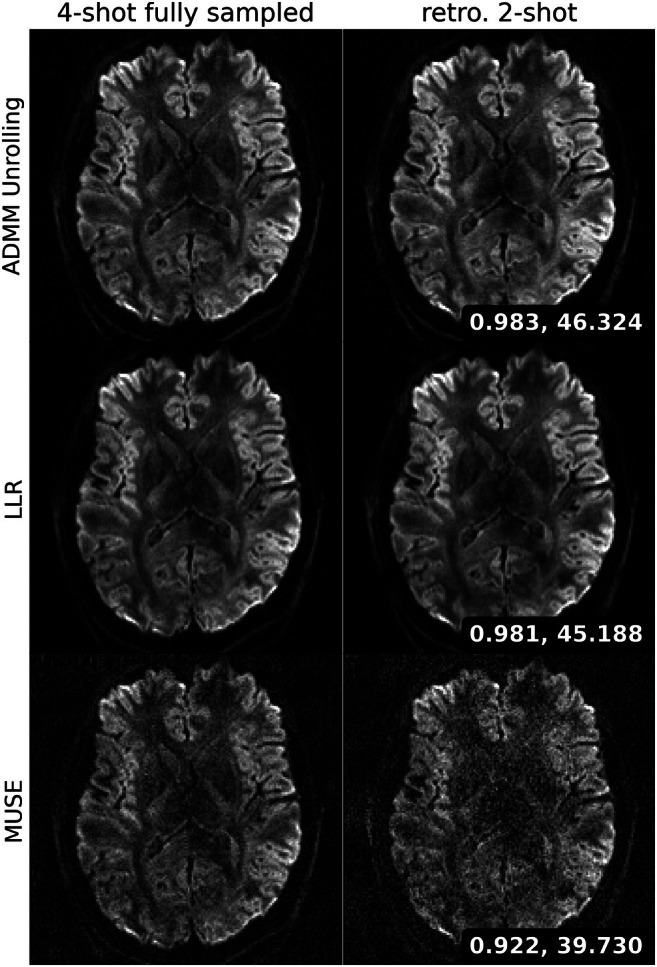
Retrospective study with the fully‐sampled reference data acquired by Protocol #1. The first column displays one diffusion‐weighted image from the 4‐shot fully‐sampled data reconstructed via (from top to bottom) the proposed self‐supervised ADMM unrolling, LLR, and MUSE. The second column displays the diffusion‐weighted image from the retrospectively undersampled 2‐shot data reconstructed via the aforementioned methods. Two image metrics, structural similarity index measure (SSIM) and peak signal‐to‐noise ratio (PSNR) are computed between the 4‐shot and the 2‐shot reconstructions.

Figure 3 validates the proposed self‐supervised ADMM unrolling reconstruction method with the 4‐shot fully‐sampled 1.0 mm dataset (Protocol #1 in Table 1). MUSE on the 2‐shot undersampled data exhibits noticeable image quality degradations, as confirmed by the visual inspections as well as the SSIM and PSNR quantities. Both LLR and ADMM unrolling are capable of reconstructing high‐quality diffusion‐weighted images without significant loss of image details and SNR. The computed quantitative metrics show ADMM unrolling performs slightly better than LLR.

In addition, an ablation study that replaces the ResNet with Identity is provided in Figure S2. Through this comparison, we observe effective denoising and sharp diffusion‐weighted contrast with the ResNet‐embedded ADMM unrolling.

### 3.2 Model Generalizability

#### 3.2.1 Cross Slices

**FIGURE 4:**
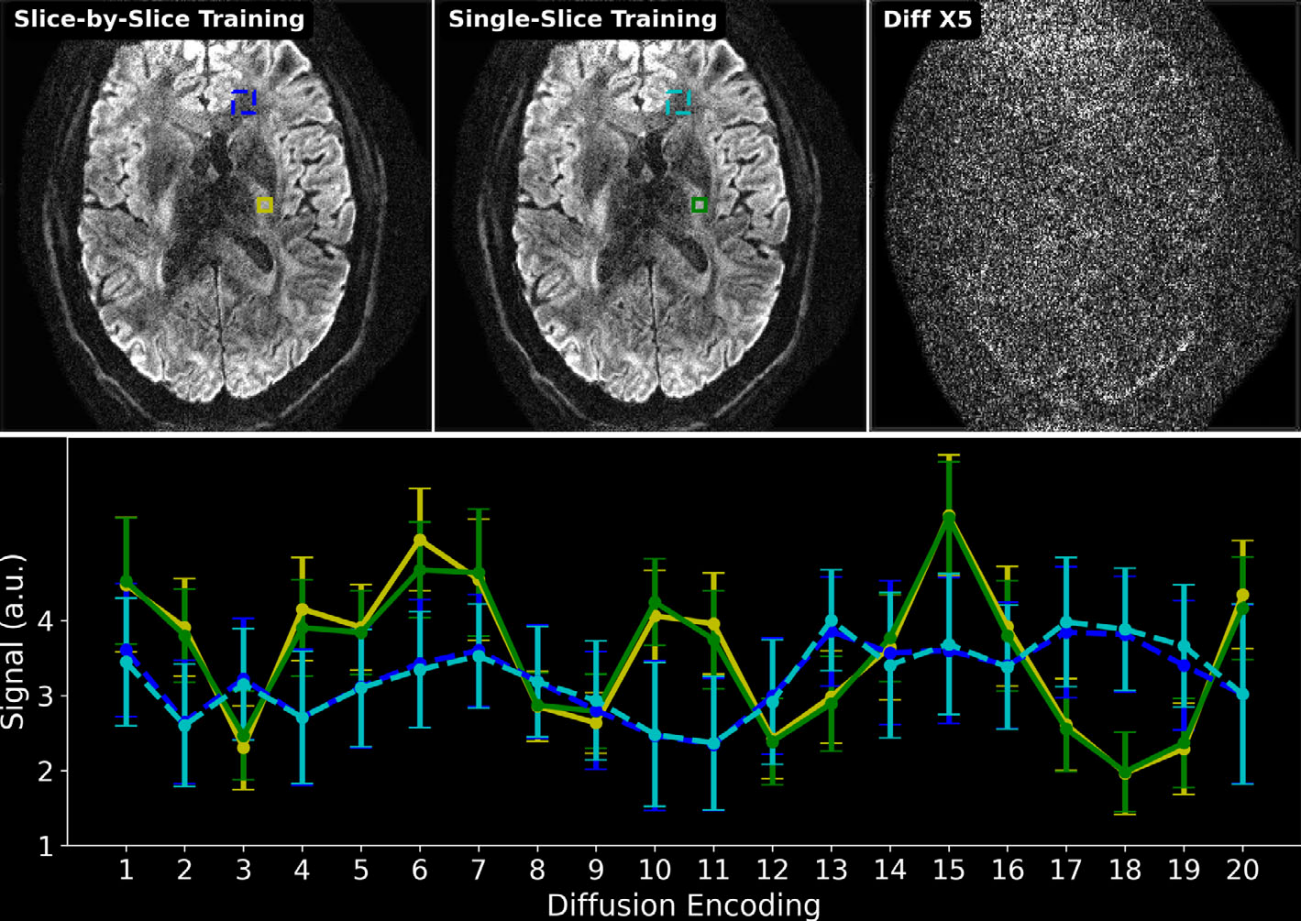
Comparison of two training strategies: (1) slice‐by‐slice training, where every slice is trained and tested individually; (2) single‐slice training, where the unrolled ADMM model is trained on only one slice and tested on all remaining slices. The top‐right image shows the absolute difference between the reconstructed diffusion‐weighted images at the 10th diffusion direction between (1) and (2). The bottom panel plots the mean and standard deviation of the signal within two sets of rectangles in the slice‐by‐slice training and the single‐slice training, respectively. No major qualitative or quantitative difference can be seen between the two training strategies.

Figure 4 demonstrates the generalizability of the proposed ADMM unrolling approach, that is, an unrolled ADMM model trained on one single multi‐band slice is applicable to all remaining “unseen” slices. Single‐direction diffusion‐weighted images from both the slice‐by‐slice training and the single‐slice training strategies are displayed. The absolute difference between these two images shows no residual structural information, but mainly noise. Moreover, we plotted the mean and standard deviation within the selected region‐of‐interest (colored boxes in Figure 4) along all diffusion encoding directions. This again proves the cross‐slice generalization of the proposed self‐gated self‐supervised ADMM unrolling method. The plotted curves show quantitatively similar values between the two training strategies. With this, the following results were obtained based upon the single‐slice training strategy.

#### 3.2.2 Cross Subjects

We also evaluate the cross‐subject generalizability. Figure S3 displays the results of a model trained on one subject but inferred on a different subject. The model performs well and is applicable to different subjects with the same acquisition parameters.

### 3.3 Self‐Gated ADMM Unrolling

**FIGURE 5:**
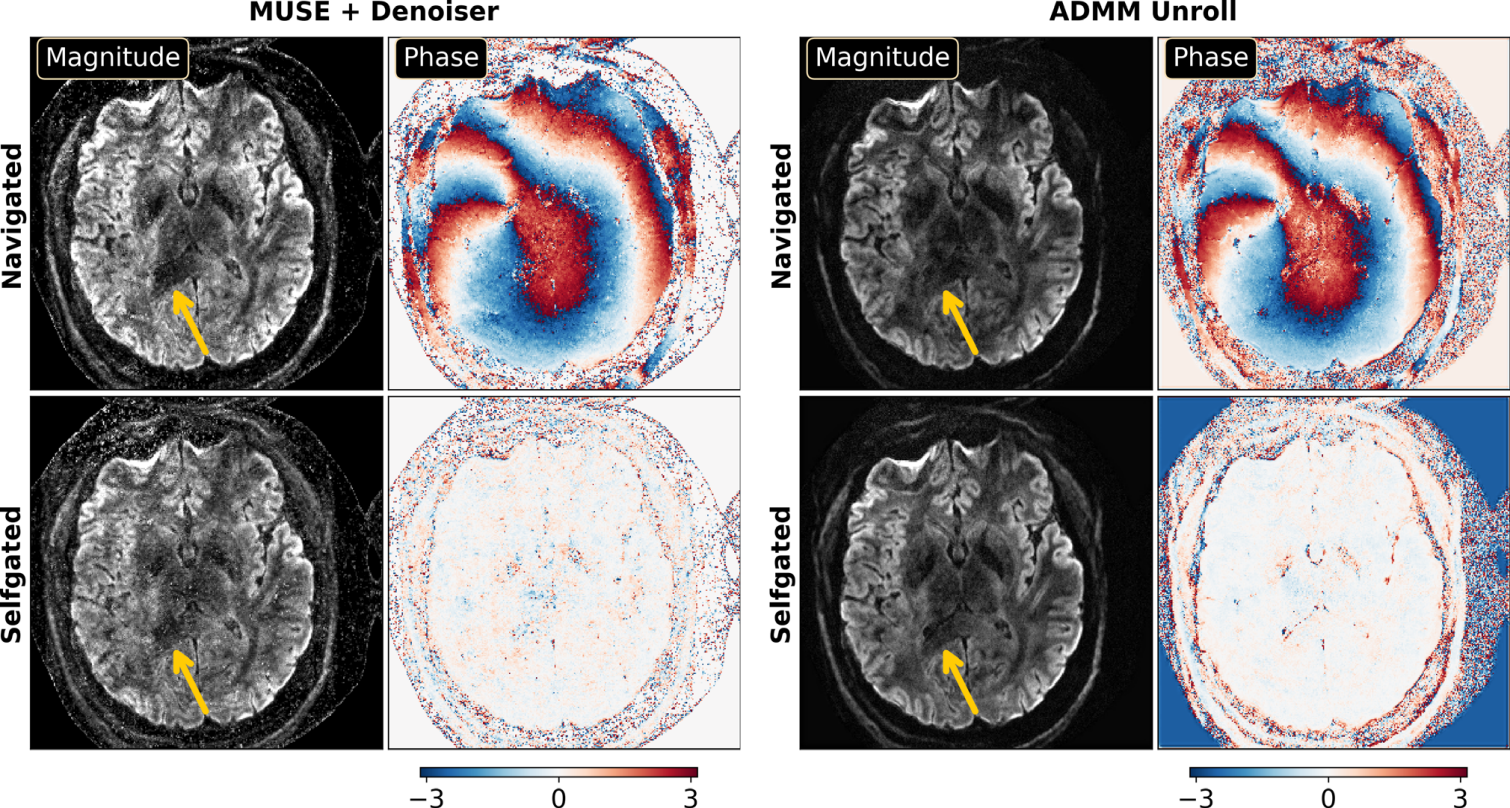
Validation of the proposed self‐gated ADMM unrolling reconstruction with data acquired by Protocol #2 in Table 1. Both MUSE and ADMM unrolling were performed with navigated and self‐gated shot‐to‐shot phase maps, respectively. Comapred to MUSE, the ADMM unrolled reconstruction excels in denoising while maintaining structural details. Self‐gated ADMM unrolling shows improved image quality in terms of tissue delineation than navigated reconstruction.

Figure 5 demonstrates the efficacy of the self‐gated self‐supervised ADMM unrolling reconstruction by comparing with the navigated reconstruction on the first volunteer. Both MUSE and ADMM unrolling reconstructions were performed. Data were acquired using the NAViEPI sequence, as listed in Protocol #2 in Table 1. The single‐direction diffusion‐weighted images are displayed.

The diffusion‐weighted images from navigated reconstructions show spatially varying phase. The reason is that the shot‐to‐shot phase variations were estimated from the second echo in NAViEPI, that is, the navigator, whose echo time is different from the first echo. The echo time difference results in residual phases in the combined diffusion‐weighted images. On the contrary, self‐gated reconstructions show only subtle phase because shot‐to‐shot phase variations were estimated from the first echoes themselves. The reduced phase variation in the self‐gated reconstruction leads to less phase ambiguity. This is beneficial in the ADMM unrolling reconstruction, where convolutions were performed in both the real and imaginary channels. Reduced phase ambiguity fosters the learning procedure. Consequently, compared to MUSE with the MPPCA denoiser [37], the self‐gated ADMM unrolling reconstruction achieves strong denoising and resolves clear tissue details.

**FIGURE 6:**
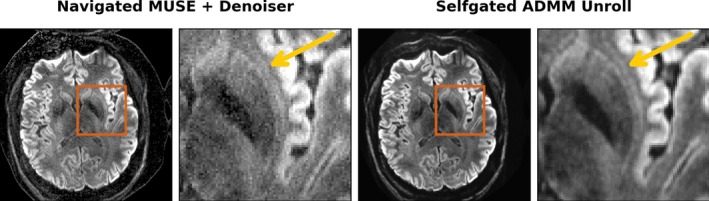
0.7 mm isotropic resolution DWI with the proposed self‐gated ADMM unrolling enables the visualization of the tiny structure claustrum, whereas the MUSE reconstruction shows only a blurred appearance. Displayed images are the mean diffusion‐weighted image from 20 directions and its zoomed‐in region.

The advantage of the proposed ADMM unrolling for high resolution DWI with accelerated acquisition is further evident in Figure 6. The mean diffusion‐weighted image from ADMM unrolling shows clear delineation of the claustrum, which is a thin sheet of neuros and is important to consciousness. In contrast, MUSE with the MPPCA denoiser shows noisy and blurred boundaries of the claustrum.

**FIGURE 7:**
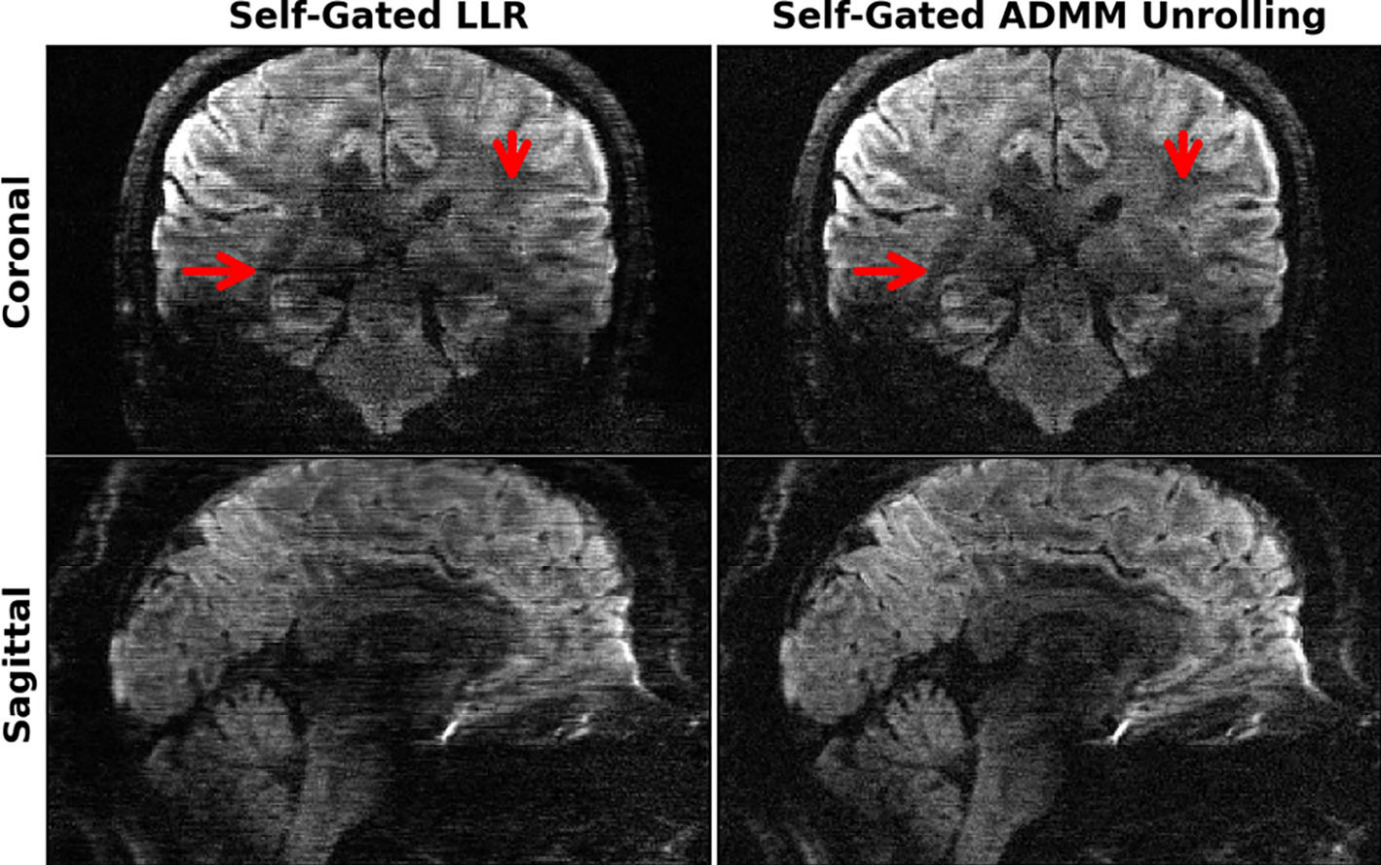
Single‐direction diffusion‐weighted images at 0.7 mm isotropic resolution as reconstructed by retrospectively self‐gated (left) LLR and (right) ADMM unrolling in (top) the coronal and (bottom) the sagittal views, respectively. The same diffusion direction as in Figure 5 is chosen for display. ADMM unrolling reduces phase ambiguities in the shot‐combined reconstruction, thereby rendering clearer tissue delineation and reducing stripping artifacts (as indicated by the red arrows).

Figure 7 shows coronal‐ and sagittal‐view diffusion‐weighted images with the same diffusion encoding as in Figure 5. As mentioned in Section 2.4, the unrolled ADMM model was trained using only one slice and then inferred on all remaining slices. Again, the single‐slice model generalizes well across slices. The inference of every slice takes only about 1 min, whereas the LLR reconstruction takes about 48 min per slice. More importantly, the self‐gated LLR reconstruction exhibits residual motion‐induced stripping artifacts [38], whereas the self‐gated ADMM unrolling approach substantially removes these artifacts and supplies high‐quality diffusion‐weighted images without the need of navigators. Both reconstructions show $B_{1}$ field inhomogeneities in the cerebellum region as well as off‐resonance induced spatial distortion in the frontal brain region. These artifacts, however, are beyond the scope of this work.

### 3.4 Diffusion Tensor Imaging (DTI)

**FIGURE 8:**
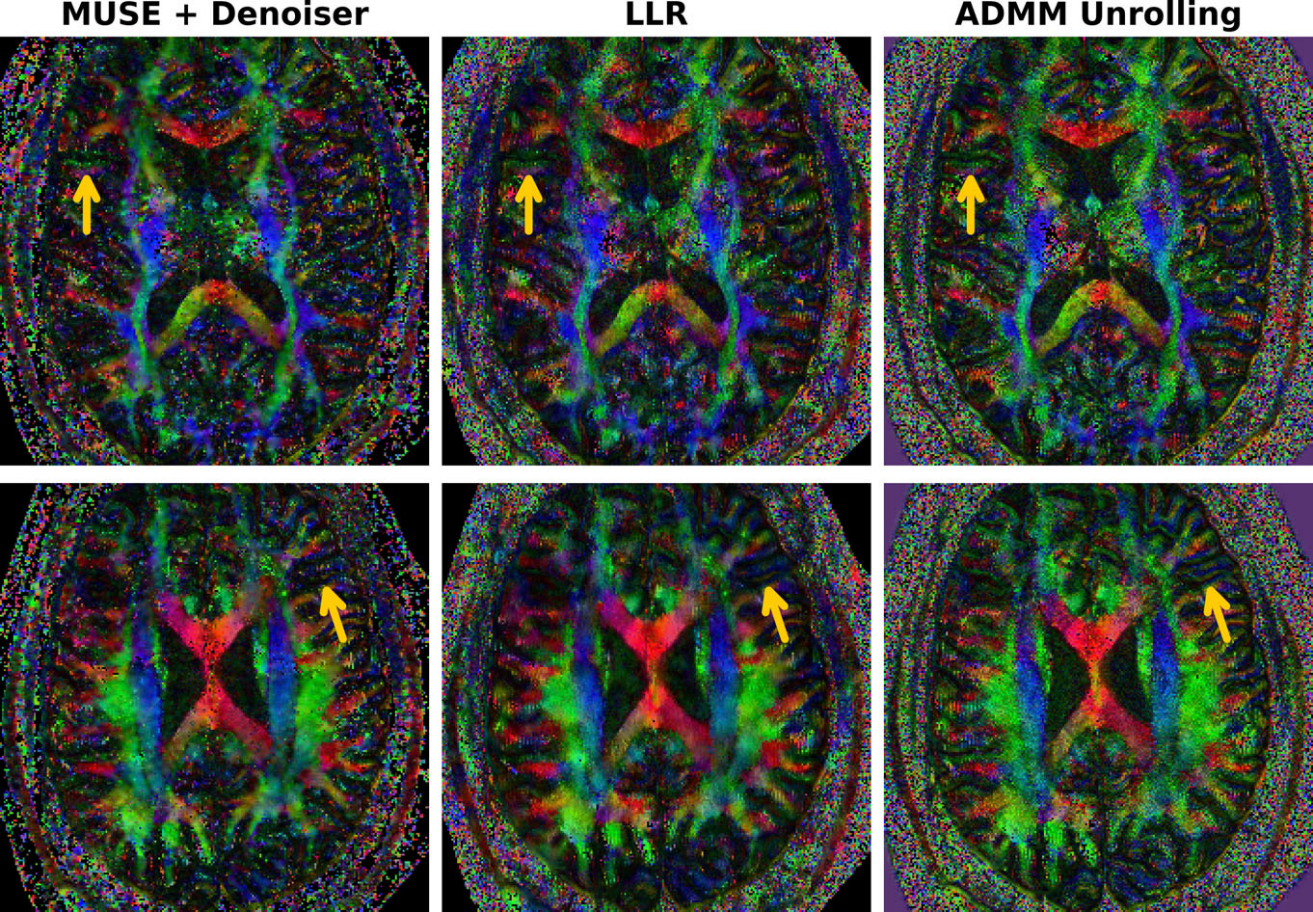
Diffusion tensor imaging (DTI) model derived colored fractional anisotropy (cFA) maps based on the diffusion‐weighted images as reconstructed by MUSE, LLR, and ADMM unrolling, respectively. Two slices are displayed in the top and the bottom row, respectively. Even with limited scan time (about 10 min at 0.7 mm spatial resolution) and limited diffusion directions (20), the proposed self‐gated self‐supervised ADMM unrolling reconstruction delivers clearer fiber orientations, as indicated by the maize‐color arrows.

Figures 4 and 8 utilize the Protocol #3 acquired data from the same volunteer. Here, Figure 8 displays the cFA maps based on the reconstructed diffusion‐weighted images by MUSE with denoiser, LLR, and ADMM unrolling, respectively. Given the 2×2‐fold acceleration and the submillimeter spatial resolution (0.7 mm), the MPPCA denoiser applied onto MUSE is insufficient to supply sharp fiber orientations. Although LLR shows improvements when compared to the MUSE approach, but still shows overall blurring in the cFA map, especially within the gray matter region. The proposed self‐gated self‐supervised ADMM unrolling is able to resolve thin fibers within gray matters, as pointed by the color arrows.

**FIGURE 9:**
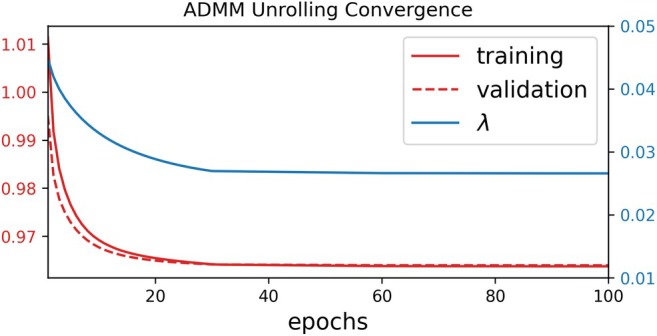
Convergence analysis along the ADMM unrolling training and validation epochs. Displayed curves are (red solid) the training loss, (red dashed) the validation loss, and (blue solid) the learned regularization strength λ, respectively. All parameters converge sufficiently and show no over‐fitting.

Figure 9 displays the training and validation loss as well as the learned regularization strength along epochs. It can be seen that 100 epochs are sufficient for the convergence of ADMM unrolling. The model converges well along epochs and does not show any overfitting behavior (the validation loss decays similarly to the training loss). In addition, the regularization strength converges to the value of about 0.027. Note that the validation loss is slightly lower than the training loss. This is because more data is split into the training mask than the validation mask.

## 4 Discussion

This work reports a novel self‐gated self‐supervised learning approach based on ADMM unrolling for multi‐shot multi‐band undersampled iEPI acquisition and high‐resolution DWI reconstruction. The self‐gated ADMM unrolling achieves whole brain DWI with 21 diffusion volumes and a b‐value of 1000s/mm 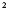 at 0.7 mm isotropic resolution, all within a scan time of less than 10 min. Our proposed ADMM unrolling approach has several advantages. (1) Inline with the previous approaches for single image recovery [31] and parallel imaging [28], our approach trains an unrolled reconstruction network with only one dataset utilizing the concept of data splitting [28, 31, 35]. Therefore, our approach is scan‐specific and does not require large‐scale datasets for training. (2) Our approach explores the joint k‐q‐space redundancy with the use of spatial‐diffusion convolutions and is also constrained by the physics‐based data consistency. Therefore, our approach is versatile to downstream diffusion model analysis (e.g., DTI). (3) We observe that the ADMM unrolling model can be trained from one single multi‐band slice and is generalizable to other “unseen” multi‐band slices. This substantially reduces the required training time. Furthermore, given that unrolled reconstructions require much shorter inference time than conventional iterative regularized reconstructions such as compressed sensing (refer to Table S1), our approach is feasible for clinical translation.

This work demonstrated the capability of self‐gated ADMM unrolling in reconstructing 0.7 mm isotropic resolution 3‐shot iEPI DWI with (6×2)‐fold acceleration per shot. For the 0.7 mm resolution with 3 shots, as shown in this work, the self‐gated acquisition is beneficial of reducing scan time, given the superior performance of the proposed ADMM unrolling reconstruction. Alternatively, employing optimized trajectories with a more densely sampled k‐space central region could help better estimate shot phase variations [4].

We observed that stripping‐type motion artifacts occurred more frequently in the sub‐millimeter isotropic resolution DWI regime. This makes sense, as scans with reduced slice thickness are more susceptible to shot‐to‐shot phase variations. In addition, sub‐millimeter isotropic voxel resulted in higher noise in diffusion‐weighted images. Since the primary aim of this work is to develop an efficient self‐supervised learning technique for sub‐millimeter DWI, we did not explore other advanced sampling strategies such as gSlider. However, because unrolled algorithms are flexible to MR physics modeling (e.g., the forward operator 𝒜), the proposed ADMM unrolling is extendable to incorporate with the gSlider encoding model for enhanced SNR performance.

This work does have several limitations. (1) This work did not incorporate off‐resonance correction in the reconstruction. As a logic extension, the multi‐shot sequence can be modified to encode dynamic $B_{0}$ field variation, which can then be employed in the SENSE‐based forward operator and reconstruction. An established approach is known as the blip‐up/down encoding [39]. This approach requires the acquisition of two images with opposing phase‐encoding polarities (i.e., blip‐up and blip‐down) for the computation of $B_{0}$ field maps. An alternative approach is to iteratively update $B_{0}$ field based on the phase difference among acquired multiple echoes [40]. This approach does not require the pre‐determination of $B_{0}$ field, but poses higher computational demand in the inversion course of phase increments from every echo. (2) As this work primarily focused on the development of self‐gated self‐supervised unrolled reconstruction for high‐resolution DWI, only three volunteers were recruited. A pilot study with a large number of volunteers and even patients is beyond the scope of this work. (3) Given the small sample size, it is unlikely that we compare our proposed approach with other semi‐self‐supervised approaches such as SSDU [35]. Only MUSE and LLR are chosen for comparison in this work. However, we believe that MUSE and LLR are competitive and representative methods to be compared with, as the former one has already been translated to clinical practice and the latter one has been widely used for multi‐contrast compressed sensing image reconstruction.

## 5 Conclusions

In this work, we proposed a self‐gated self‐supervised learning reconstruction framework based on ADMM unrolling for high‐resolution and motion‐robust DWI at ultra‐high field. Based on the mechanism of data splitting (cross validation), our proposed ADMM unrolling requires only one single multi‐band slice for training and is generalized cross‐slice. Plus, ADMM unrolling renders ultra‐short inference/reconstruction time and is thus feasible for clinical translation.

## Funding

This work was supported by German Research Foundation (DFG) projects 513220538, 512819079, and project 500888779 in the Research Unit RU5534 for MR biosignatures at UHF. National Institutes of Health (NIH) grants R01EB024532 and P41EB017183.

## Conflicts of Interest

Patrick A. Liebig is an employee of Siemens Healthcare GmbH. Florian Knoll receives research funding from Siemens Healthineers AG; patent royalties for AI for MR image reconstruction from Siemens Healthineers AG; holds stock options from Subtle Medical Inc.; serves as scientific advisor to Imaginostics Inc.

## Supporting information


**Data S1:** mrm70250‐sup‐0001‐Supinfo.pdf.

## Data Availability

In the spirit of open science and reproducible research, source codes of this work are available in https://github.com/ZhengguoTan/DeepDWI. The presented 0.7 mm DWI raw k‐space data is available in https://doi.org/10.5281/zenodo.10781347, https://doi.org/10.5281/zenodo.17618072, and https://doi.org/10.5281/zenodo.13864504.
