# Supplementary material for: High‐Resolution Diffusion‐Weighted Imaging With Self‐Gated Self‐Supervised Unrolled Reconstruction
Source: Magn Reson Med. 2026 Jan 22;95(5):2852–62. doi: 10.1002/mrm.70250 (PMC12962217; doi:10.1002/mrm.70250)
Supplement: Supplementary file 1 — Data S1: mrm70250‐sup‐0001‐Supinfo.pdf. [file MRM-95-2852-s001.pdf]

Supplementary Information

High-Resolution Diffusion-Weighted Imaging with Self-Gated Self-Supervised Unrolled Reconstruction

Zhengguo Tan, Patrick A. Liebig, Annika Hofmann, Frederik B. Laun, Florian Knoll

1 Residual Neural Network (ResNet) 2

2 Ablation Study 3

3 Generalizability: Cross Subjects 4

4 Reconstruction Time 6

1 Residual Neural Network (ResNet)

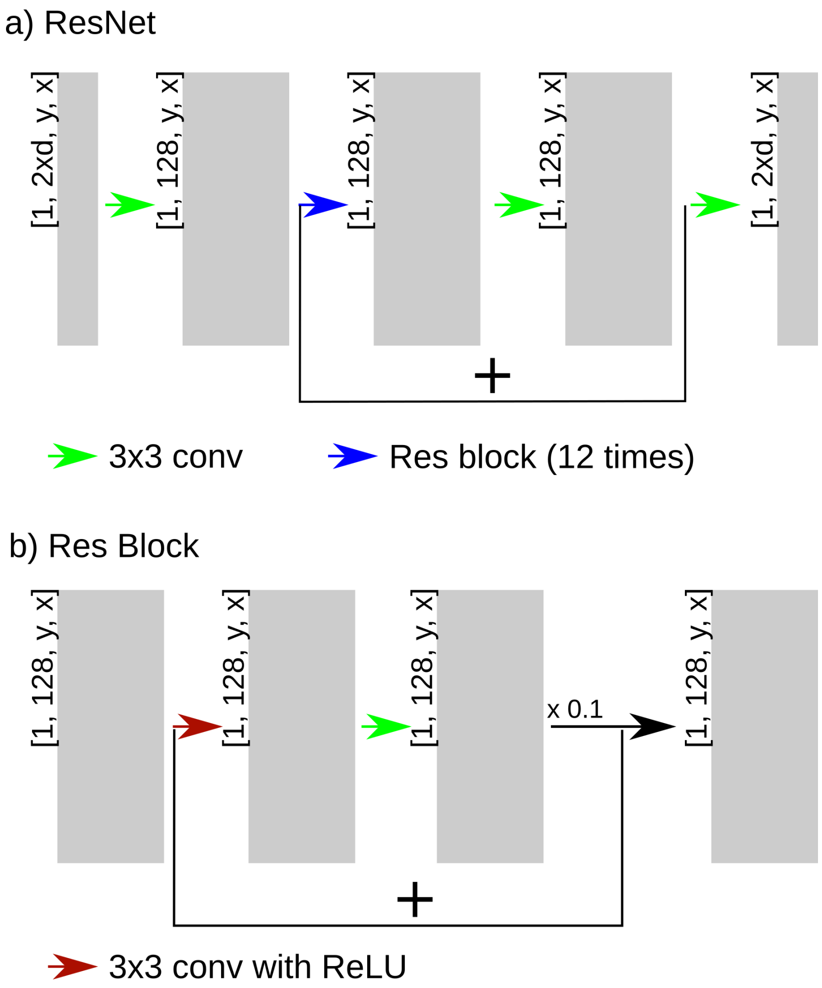

Figure S1: The architecture of ResNet. x and y denotes the shape of diffusion-weighted images, d denotes the number of diffusion encodings, and 2 indicates the real and the imaginary part.

In this study, the acquisition base resolution is 286, i.e., $x = y = 286$. The ResNet architecture in Figure S1 consists of $3,786,539$ trainable parameters.

2 Ablation Study

In machine learning, ablation means the removal of a component of an artificial intelligence (AI) system. Here, we replace the ResNet with an Identity module (torch.nn.Identity()), which returns its input without any modification or computation, and has no trainable parameter. As a reuslt, only the regularization parameter λ remains trainable during ADMM unrolling.

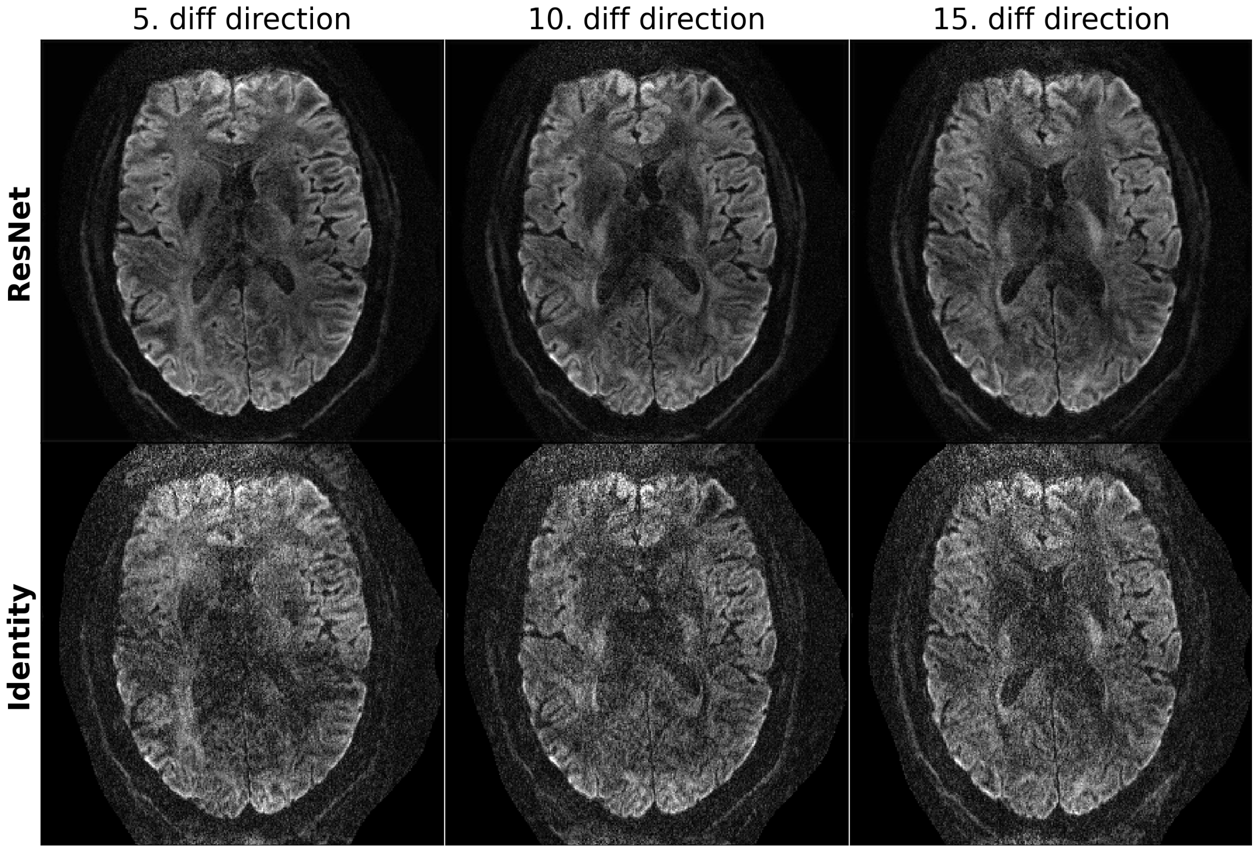

Figure S2: Ablation study. (1st Row) Reconstruction results of the proposed ADMM unrolling with the ResNet architecture. (2nd Row) Reconstruction results of ADMM unrolling without ResNet, but the Identity operator as the regularization.

Figure S2 displays the reconstruction results of the designed ablation study. This ablation study demonstrates the important role of the learned ResNet as a regularizer in removing noise while retaining sharp diffusion-weighted contrasts.

3 Generalizability: Cross Subjects

While Figure 4 in the main text shows the cross-slice generalizability of the proposed ADMM unrolling method, here we try to investigate whether the method generalizes among subjects. Specificially, we use the checkpoint from Figure S2 for the reconstruction of the data in Figure 4.

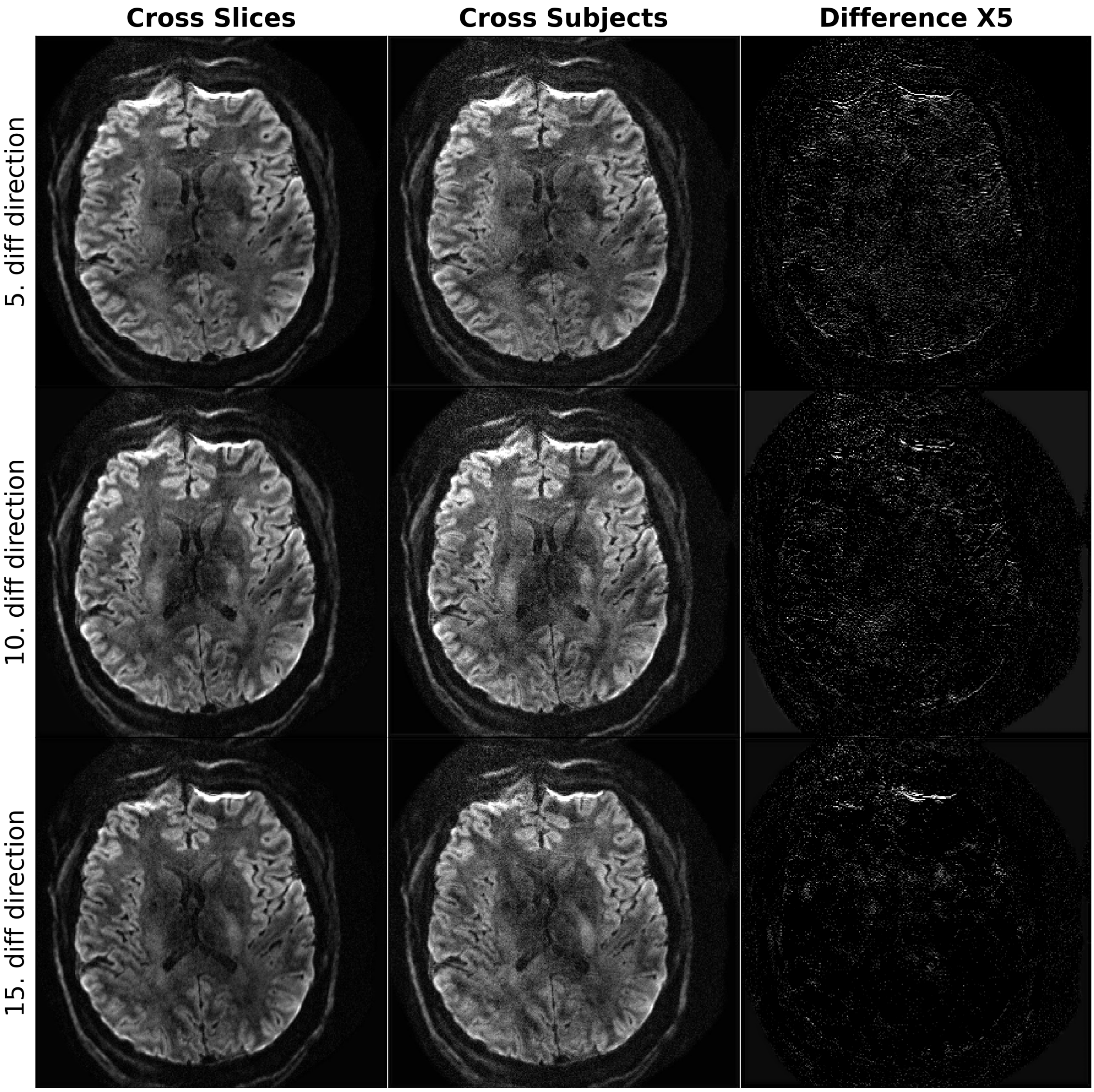

Figure S3: Cross-subject generalizability. (1st Column) Training and inference with the same subject, but only one slice is used for training. (2nd Column) Training and inference with difference subjects. Here, training is performed with the data in Figure S2, whereas inference is performed on a different subject. (3rd Column) Subtration of diffusion-weighted images in the 1st column from those in the 2nd column.

Figure S3 shows the results of the cross-subject generalizability study. The difference images show only subtle differences between the two reconstruction results: cross-slice and cross-subject inferences. The proposed ADMM unrolling method generalizes well cross slices and subjects. We

foresee that ADMM unrolling with the self-supervised training can serve as a generalized model for diffusion-weighted image reconstruction and for fast inference.

4 Reconstruction Time

Table S1: Comparison of reconstruction times per multi-band slice. All reconstructions were performed on GPU A100 with 80GB memory.

Method	Training time (h)	Inference time (min)
MUSE	-	02:08
LLR	-	54:00
ADMM Unroll	04:27	01:50
